# Supplementary material for: Shaping exciton polarization dynamics in 2D semiconductors by tailored ultrafast pulses
Source: Light Sci Appl. 2025 Feb 11;14:80. doi: 10.1038/s41377-025-01748-7 (PMC11814118; doi:10.1038/s41377-025-01748-7)
Supplement: Supplementary file 1 — Supplementary information [file 41377_2025_1748_MOESM1_ESM.docx]

Supplementary Information for –

Shaping Exciton Polarization Dynamics in 2D Semiconductors by Tailored Ultrafast Pulses

Omri Meron^1,2^, Uri Arieli^1,2^, Eyal Bahar^1,2^, Swarup Deb^1^, Moshe Ben-Shalom^1^ and Haim Suchowski^1,2†^

^1^Condensed Matter Physics Department, School of Physics and Astronomy, Faculty of Exact Sciences, Tel Aviv University, Tel-Aviv, 6997801, Israel.
^2^Center for Light-Matter Interaction, Tel Aviv University, Tel-Aviv, 6997801, Israel

##

Table of Contents

[S1 Photoluminescence Measurements 2](#_Toc180857564)

[S2 Single-pulse nonlinear wave-mixing 2](#_Toc180857565)

[S2.1 Intra-pulse sum frequency generation (SFG) 2](#_Toc180857570)

[S2.2 Intra-pulse four-wave mixing (FWM) 2](#_Toc180857571)

[S2.3 Instantaneous Vs. noninstantaneous nonlinear wave-mixing 4](#_Toc180857572)

[S2.4 Detecting resonant dynamics via. SFG 4](#_Toc180857573)

[S3 Estimation of the exciton density 6](#_Toc180857574)

[S4 Error estimation 6](#_Toc180857575)

[S5 Theoretical modeling – 3^rd^ order nonlinearity 7](#_Toc180857576)

[S5.1 TMD Bloch equations of motion 7](#_Toc180857577)

[S5.2 Damped Duffing Oscillator 9](#_Toc180857578)

[S5.3 Two-Level System (TLS) 10](#_Toc180857579)

[S6 Control of nonlinear wave-mixing – multiphoton pathway interference 12](#_Toc180857580)

[S6.1 FWM destruction 12](#_Toc180857581)

[S6.2 Geometrical representation 13](#_Toc180857582)

[S7 The relative contribution of exciton-exciton interactions and Pauli blocking to FWM 14](#_Toc180857583)

[S8 Amplitude scan of the 2s arctangent phase 15](#_Toc180857584)

[References 16](#_Toc180857585)

## Photoluminescence Measurements

To authenticate the measurement of a monolayer of WSe_2_, we conducted a photoluminescence spectrum analysis using a 532 nm continuous wave (CW) diode laser in ambient conditions.


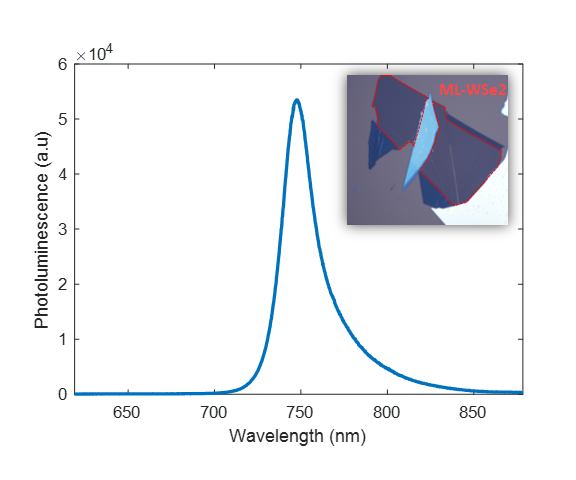


**Figure S1** **Photoluminescence spectrum of a monolayer WSe_2_ on 90nm SiO_2_ on Si substrate**. The measurement was obtained using a CW diode laser with wavelength 532 nm.

## Single-pulse nonlinear wave-mixing

The interaction between Ultrafast broadband laser pulses with a nonlinear mediator enables the generation of various intra-pulse coherent wave-mixing processes, as illustrated in Fig. [S2](#figS1).


## Intra-pulse sum frequency generation (SFG)

Intra-pulse SFG is generated by the sum of all possible photon pairs within the pulse bandwidth (see Fig.[S2](#figS1).b). For an instantaneous interaction, the SFG can be obtained by calculating the auto-convolution of the incoming electric field.

## Intra-pulse four-wave mixing (FWM)

The FWM signal refers to the nonlinear signal arising from three frequency components which sum generate a fourth component within, but also in the outskirts of the generating pulse bandwidth. For every combination of three frequency components, there are two general pathways that can generate the fourth wave (see Fig.[S2](#figS1).c). Like the SFG process, for an instantaneous interaction, the FWM spectrum can be obtained by a third-order auto-convolution of the incoming electric field (see Fig. [S2](#figS1).d). To obtain a background-free signal we usually truncate the high-frequency portion of the broadband pulse and detect the so-called ’Anti-Stokes’ FWM signal that forms near the pulse edge. Figure [S](#figS1)3 shows the cubic power dependency of the ’Anti-Stokes’ FWM. Such a third-order nonlinear signal is advantageous over other nonlinear spectroscopic techniques (such as SFG) for not being limited by symmetry constraints, by improved spatial scanning resolution, and by a relatively simple experimental setup. Such single-pulse FWM schemes have mainly been utilized for single-pulse coherent anti-Stokes Raman scattering (CARS) [1], nonlinear coherent spectroscopy for label-free detection [2] as well as other applications [3] [4].

| 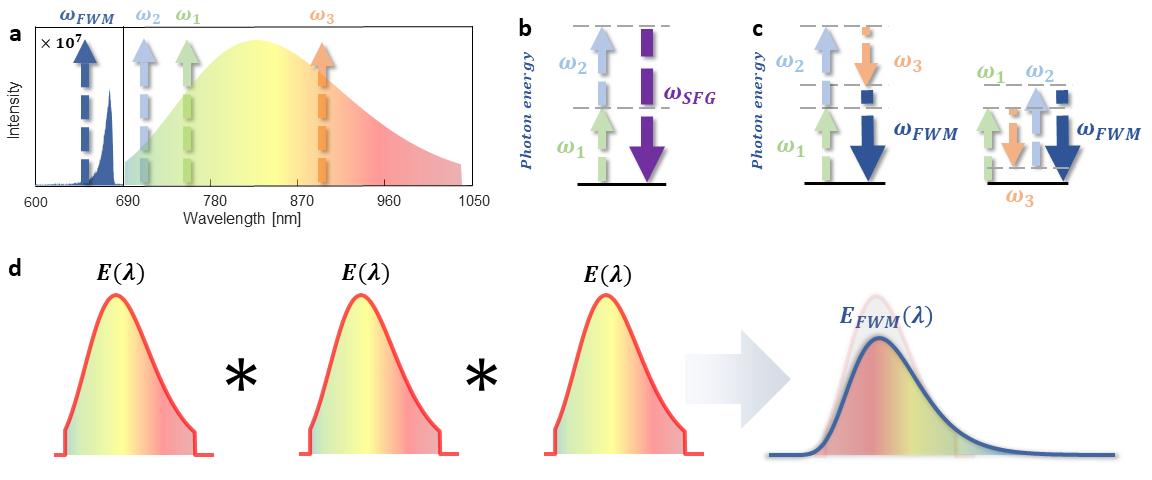 |
| --- |

**Figure S2 Single-pulse ultrafast nonlinear wave-mixing processes.** (a) Right: our ultrabroad-band laser spectrum (690-1030 nm). Left: The nonlinear four-wave mixing (FWM) spectrum (600-690 nm). (b-c) Schematic illustration of two selected wave-mixing processes. Each process illustrates a superposition of photons from within our ultra-broadband pulse spectrum (the arrows are colored in correspondence with the laser spectrum from (a)). (b) Sum frequency generation (SFG). (c) FWM.


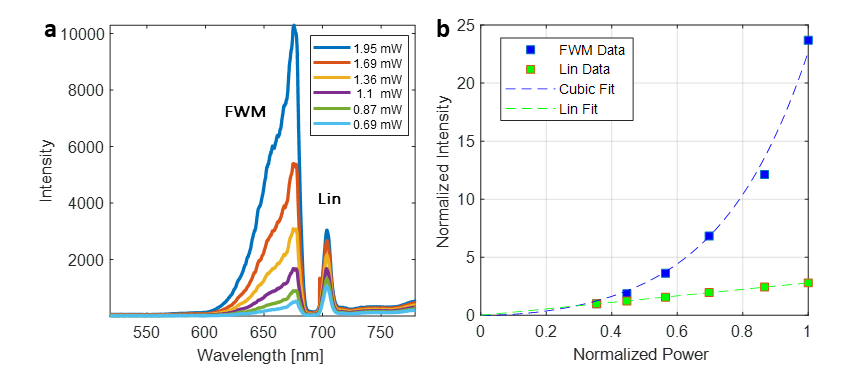


**Figure S3 Cubic power dependency of Single-pulse FWM.** (a) A measured spectrum containing a FWM signal (<680 nm) and a remnant of the linear signal (>690 nm) from a LiNbO_2_ crystal after short-pass filtration, plotted for different excitation powers. (b) Power-law dependencies of FWM and Linear signals from (a).

## Instantaneous Vs. noninstantaneous nonlinear wave-mixing

In the realm of nonlinear light-matter interactions, the nonlinear generation is essentially an instantaneous process, except for situations where a resonance occurs. This temporal photo-induced response can be studied by varying the pulse group delay dispersion (GDD) using a parabolic spectral phase that linearly changes the instantaneous frequency of the pulse, also known as a linear chirp [5] (see the illustration in main text Fig. 3.a for a linear chirp phase). In an Instantaneous process, one would expect the maximal nonlinear signal to be generated by a transform-limited (TL) pulse, as seen from SFG and FWM nonlinear signals in Fig. S4.


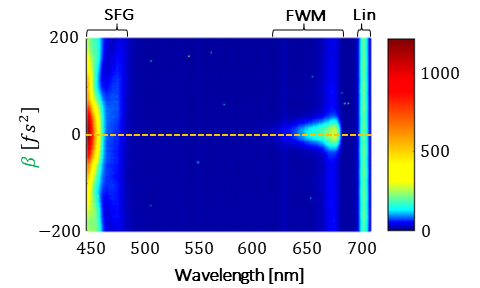


**Figure S4 GDD scan on a LiNbO3 crystal.** A measured spectrum of the intrapulse wave mixing signal as a function of the linear chirp (GDD) applied by the SLM. The linear component (>690 nm) of the spectrum shows no dependence on the pulse-shape, in contrast to the strong dependence displayed by SFG (<500 nm) & FWM (600-680 nm) nonlinear signals.

As mentioned in the main text, simple resonant dynamics exhibit a temporally asymmetric noninstantaneous response originating from a $\pi$-phase shift around the resonant frequency. In such cases, provided that the incoming pulse duration is shorter than the resonant decoherence time scale, the maximal degree of nonlinear yield may not necessarily be achieved by TL pulses (as can be seen from Fig.2.a in main text) [6]. This noninstantaneous resonant effect has been observed in SFG while exciting gold split ring resonators on resonance [5], and in this work by observing the FWM response (see Fig. 2.a in the main text).

## Detecting resonant dynamics via. SFG

The measured GDD scan of a monolayer WSe_2_ (Figure S5.c) shows the same characteristics of an instantaneous non-resonant response measured using a BBO crystal (Figure S5.a). These two measurements, performed under the same ambient conditions, match the predicted instantaneous SFG response calculated far from resonance (Figure S5.b), and differ from the case of a non-instantaneous response, presented by Fig. S5.d, and calculated using an anharmonic oscillator (using the same resonant parameters as the A:1s exciton while excited on resonance). The SFG measurements were obtained using our sub-10fs laser pulse polarized along the armchair crystallographic axis (see section ‎S3 for the estimated exciton energy). In addition, the simulations displayed in Fig. 3 take into account an attenuation factor which describes the inefficient transmission and detection of our specific experimental setup approaching the UV spectral range.


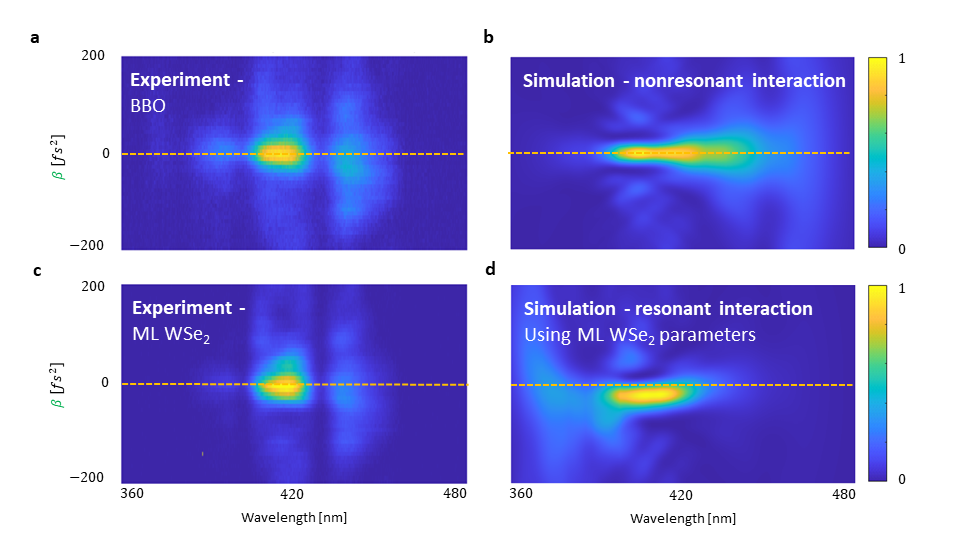
**Figure S5 Measurements and simulations of the SFG spectrum generated by a chirped pulse, with a varying GDD of β.** a-b) Experiment and Numerical simulation (respectively) of the instantaneous (far from resonance) SFG response generated by a BBO crystal. c) The SFG spectrum generated by a WSe_2_ monolayer. d) a numerical simulation of a non-instantaneous (resonant) response carried out using the anharmonic oscillator model with the A:1s resonance parameters.

Another indication for the instantaneous-like response of the WSe_2_ monolayer can be found in Fig. S6. In Fig. S6.a we present the SFG intensity measured as a function of an arctangent phase while varying the resonant frequency $\Omega$ (a similar method as presented in Fig. 3.e in the main text). These experimental results resemble the numerical simulation calculated far from resonance, see Fig. S6.b, and substantially differ from the simplest resonant case of an anharmonic oscillator presented in Fig. S6.c. This result can either indicate that the resonant $\chi_{R}^{\left( 2 \right)}$contribution we expect to measure seems to be screened by a much more dominant non-resonant $\chi_{NR}^{\left( 2 \right)}$contribution, or that a much more complex multiphoton interference effect is taking place.


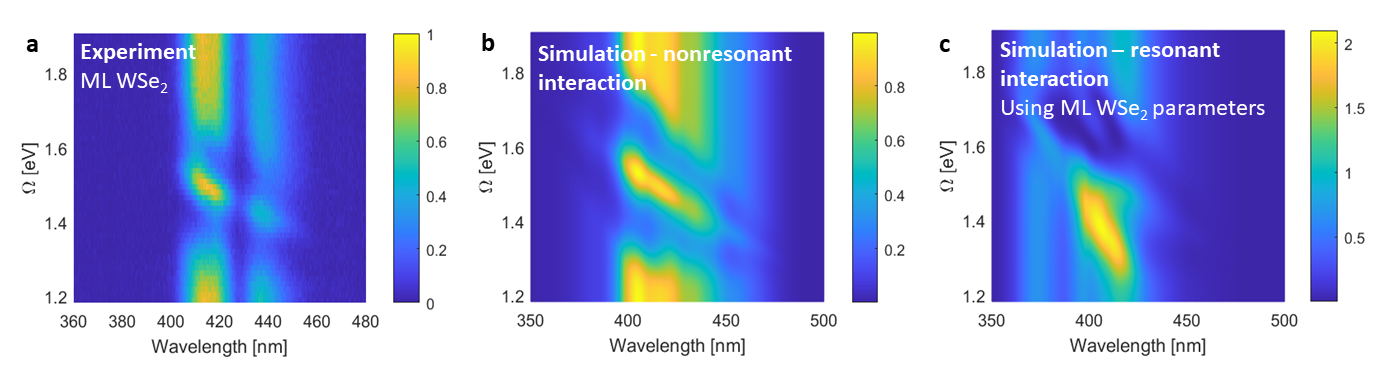
**Figure S6** **Measurements and simulations of the SFG spectrum generated by an arctangent phase scan, with a varying central frequency of Ω .** a) measurement of a monolayer WSe_2_ crystal. b-c) Numerical simulation using the anharmonic oscillator model while exciting far from resonance (instantaneous) and on resonance (non-instantaneous) respectively (The $\gamma$ parameter dictating the width of the Arctangent phase was held equal to 32 meV same as for the FWM measurements from Fig. 3 in the main text).

## Estimation of the exciton density

The pump power we have used for FWM generation was 40 μW (65 μW for SFG), which corresponds to a pump fluence of 140 $\mu J cm^{-2}$, meaninga photon density of $5.8\times{10}^{14}$ $cm^{-2}$ ($9.4\times{10}^{14}$ $cm^{-2}$ for SFG). However, since our ultra-broadband pulse is much broader than the exciton resonance, we assume only 31% of the pulse, the fraction within a spectral range of $\pm5\gamma_{X}$ from the exciton resonance $\omega_{X}$ can be accounted for exciting an exciton. Taking an approximate average absorbance of $1\%$, we estimate the exciton density to be $1.8\times{10}^{12} cm^{-2}$ ($2.9\times{10}^{12} cm^{-2}$ for SFG).

## Error estimation

The typical errors for determining the maximal GDD scan can stem from three main sources:

1. Inaccuracies in the SLM's response to a certain phase curve. Such Inaccuracies stem from the wavelength-dependent phase response of the SLM. To quantify this inaccuracy, we have conducted a set of GDD scans with a varying constant phase $\phi_{c}$ (see Figure 1), thus scanning all possible phase values per pixel while maintaining the parabolic phase trend (GDD). The maximal GDD shift we have observed for a FWM measurement on a BBO crystal is at most $\pm2 fs^{2}$.

**Figure S7 GDD scans for different constant phases along the SLM.** The color bar corresponds to the FWM intensity.

1. Errors in the dispersion pre-compensation process used to determine the transform-limited pulse. Dispersion compensation is crucial for accurate measurements and achieved through various methods.
   1. The use of a chirp mirror pair (5 double bounces on DCM-11 GVD-Mirror Pair).
   2. Rough GDD compensation by varying the length of the 4f system (grating position in the pulse shaper).
   3. Delicate compensation by applying a polynomial phase to the SLM with higher order terms (TOD *etc.*).

The correct compensation is determined when the SFG and FWM in a GDD scan using a BBO are centered at a value of $0\pm2 fs^{2}$.

1. Laser Power fluctuations. Small fluctuations in the laser power become significant in the FWM due to the cubic relation with the electric field and may affect the accumulated error with respect to the measurement units, and resolution. In the GDD scan the error is estimated to be $\pm6 fs$.

In the arctangent phase scans the estimated accuracies are $\Delta\omega_{X}=\pm4.6 meV$ and ${\Delta\gamma}_{X}=\pm3 meV$ .”

## Theoretical modeling – 3^rd^ order nonlinearity

## TMD Bloch equations of motion

We base our analysis on the equation of motion (EOM) derived by A. Knorr et.al and use them to extract the third-order parametric nonlinearity [7] [8] [9]. To simplify the analysis of the full Bloch equations of motion we added some reasonable assumptions for a weak near-infrared excitation in ambient conditions. First, we neglect the contribution of all exciton levels above A:1s. Second, we disregard incoherent terms since we measure a parametric process. Third, we neglect the contribution of exciton coupling to higher order bound states such as Biexcitons etc.

The equation of motion for the A:1s-excitons becomes:

1. $\left( \partial_{t}+\gamma_{X}-\frac{i}{\hbar}\epsilon_{X} \right)p=i\frac{d}{\hbar}E^{*}\left( t \right)+\frac{i}{\hbar}\hat{d}E^{*}\left( t \right)p\left( t \right)p^{*}\left( t \right)+\frac{i}{\hbar}\hat{V}p\left( t \right)p^{*}\left( t \right)p\left( t \right)$,

where $p\left( t \right)$ is the ground state to A:1s dipole transition which is calculated from the off-diagonal term in the density matrix representation. $\epsilon$ is the exciton energy, $\gamma$ the dephasing rate, $d$ the optical transition matrix element and $E\left( t \right)$ the electric field at the position of the monolayer. The second term on the right-hand side (RHS) characterizes a third-order contribution due to Pauli blocking (PB) by coherent excitons. The third term on the RHS of Eq. ([S1](https://www.nature.com/articles/s41467-020-18835-5#Equ2)) characterizes third-order contribution due to nonlinear exciton–exciton (X-X) interactions.

The linear solution is:

$$\tilde{p}_{0}\left( \omega\right)=-\frac{d\tilde{E}^{*}\left( -\omega\right)}{\hbar}\frac{1}{\omega+\omega_{X}+i\gamma_{X}}$$

while we Set $\epsilon_{X}=\hbar\omega_{X}$. The total dipole moment is given by the sum of $p\left( t \right)$ and $p^{*}\left( t \right)$:

$$\tilde{P}\left( \omega\right)=\tilde{p}_{0}\left( \omega\right)+\tilde{p}_{0}^{*}\left( -\omega\right)=-\frac{d\tilde{E}\left( \omega\right)}{\hbar}\frac{2\omega_{X}}{\omega_{X}^{2}+\gamma_{X}^{2}-\omega^{2}-2i\gamma_{X}\omega}$$

To extract the third-order nonlinear contribution we add a perturbation $\delta p\left( t \right)$ to the linear solution $p_{0}\left( t \right)$ and rewrite the EOMs:

$$\begin{matrix} \left( \partial_{t}+\gamma_{X}-\frac{i}{\hbar}\epsilon_{X} \right)\left( p_{0}\left( t \right)+\delta p\left( t \right) \right)=i\frac{d}{\hbar}E^{*}\left( t \right)+\frac{i}{\hbar}\hat{d}E^{*}\left( t \right)\left( p_{0}\left( t \right)+\delta p\left( t \right) \right)\left( p_{0}\left( t \right)+\delta p\left( t \right) \right)^{*} \\ +\frac{i}{\hbar}\hat{V}\left( p_{0}\left( t \right)+\delta p\left( t \right) \right)\left( p_{0}\left( t \right)+\delta p\left( t \right) \right)^{*}\left( p_{0}\left( t \right)+\delta p\left( t \right) \right) \end{matrix}$$

The EOM for $\delta p\left( t \right)$ after neglecting the weaker terms:

$$\left( \partial_{t}+\gamma_{X}-\frac{i}{\hbar}\epsilon_{X} \right)\delta p\left( t \right))=\frac{i}{\hbar}\hat{d}E^{*}\left( t \right)p_{0}\left( t \right)p_{0}^{*}\left( t \right)+\frac{i}{\hbar}\hat{V}p_{0}\left( t \right)p_{0}^{*}\left( t \right)p_{0}\left( t \right)$$

We solve the equations in Fourier space, obtaining the total dipole moment:

1. $\begin{matrix} \delta\tilde{P}\left( \omega\right)=\frac{-1}{\hbar\left( \omega+i\gamma_{X}+\omega_{X} \right)}\left( \hat{d}\tilde{E}^{*}\left( -\omega\right)*\tilde{p}_{0}\left( \omega\right)*\tilde{p}_{0}^{*}\left( -\omega\right)+\hat{V}\tilde{p}_{0}\left( \omega\right)*\tilde{p}_{0}^{*}\left( -\omega\right)*\tilde{p}_{0}\left( \omega\right) \right) \\ +c.c , \end{matrix}$

where the first RHS term is again attributed to PB, while the second term to X-X interactions.

The off-diagonal term can also be represented using [Euler's formula](https://en.wikipedia.org/wiki/Euler%27s_formula):

$$\tilde{p}_{0}\left( \omega\right)=-\frac{d\tilde{E}^{*}\left( -\omega\right)}{\hbar}\frac{1}{\omega+\omega_{X}+i\gamma_{X}}=\left| \tilde{E}^{*}\left( -\omega\right) \right|\cdot\left| \tilde{D}^{*}(-\omega) \right|e^{-i\left( \phi_{E}\left( -\omega\right)+\phi_{D}\left( -\omega\right) \right)}$$

where $\tilde{D}\left( \omega\right)=\frac{d}{\hbar}\left( \omega-\omega_{X}+i\gamma_{X} \right)^{-1} ,$The argument of the weight function $D\left( \omega\right)$ finds the form:

$$\arg\left\{ \tilde{D}\left( \omega\right) \right\}=\phi_{D}=\tan^{-1} \frac{\omega-\omega_{X}}{\gamma_{X}}$$

The simplest way to maximize the X-X interaction term in Eq. S2, including a third-order convolution of the of diagonal dipole terms would be to set its’ arguments to zero. This manipulation can be achieved by applying a complementary phase argument to the incoming electric field:

$\arg\left\{ \tilde{E}\left( \omega\right) \right\}=\phi_{E}\left( \omega\right)=-\phi_{D}\left( \omega\right)=$ $-\tan^{-1} \frac{\omega-\omega_{X}}{\gamma_{X}}$

## Damped Duffing Oscillator

The equation of motion of the damped Duffing oscillator are:

1. $\ddot{x}+2\gamma\dot{x}+\omega_{0}^{2}x+\alpha x^{3}=-\frac{eE\left( t \right)}{m}$

where $\omega_{0}$ is the resonance frequency, $\gamma$ the damping rate, $e$ the electron charge, m the reduced mass, E(t) is the driving electrical field and $\alpha$ is a nonlinear correction to the quadratic potential. By setting $\alpha$ to zero in Eq. S3 we obtain the linear oscillator displacement $x_{0}\left( t \right)$:

$$x_{0}\left( t \right)=\int\frac{e}{m}|\tilde{E}\left( \omega\right)||\tilde{D}\left( \omega\right)|e^{\phi_{E}\left( \omega\right)+\phi_{D}\left( \omega\right)-i\omega t}d\omega$$

Here $\tilde{D}\left( \omega\right)=\left( \omega^{2}-\omega_{0}^{2}-2i\omega\gamma\right)^{-1}$ signifies the stationary solution of $x_{0}(\omega)$. By inserting a small perturbation to the linear displacement $x\left( t \right)=x_{0}\left( t \right)+\delta x\left( t \right)$. The equation of motion of the perturbative term $\delta x\left( t \right)$ then takes the approximate form of:

$$\delta\ddot{x}\left( t \right)+2\gamma\delta\dot{x}\left( t \right)+\left( \omega_{0}^{2}-3\alpha x_{0}^{2} \right)\delta x\left( t \right)\approx-\alpha x_{0}^{3}\left( t \right)+O(\delta x^{2})$$

The solution in Fourier space forms the following nonlinear response:

1. $\delta x\left( \omega\right)\approx-\alpha\frac{\tilde{x}_{0}\left( \omega\right)*\tilde{x}_{0}\left( \omega\right)*\tilde{x}_{0}\left( \omega\right)}{\tilde{D}\left( \omega\right)}$

We note that among other small terms $O\left( \delta x^{2} \right)$, Eq. S4 neglects the small shift in resonance due to excitation. We justify this, as we have not experimentally observed any significant shift in the resonant frequency within the relevant range of error, and numerically, no significant difference in the resulting FWM while solving the full differential equation of motion in the time domain. Unlike similar treatment for narrow-band sources where the convolution is reduced to a sum over a few discrete mixing frequencies [10], we are interested in preserving all intra-pulse four-wave combinations within our ultra-broadband source.

From Eq. S4 we conclude the nonlinear response, $\delta x$, is maximized if $\tilde{x}_{0}\left( \omega\right)=\left| \tilde{x}_{0}\left( \omega\right) \right|$. This can be achieved by setting the electric Field phase to be:

$$\phi_{E}\left( \omega\right)=\phi_{D}\left( \omega\right)=-\tan^{-1} \frac{2\gamma\omega}{\omega^{2}-\omega_{0}^{2}}\approx-\tan^{-1} \frac{\omega-\omega_{0}}{\gamma}$$

The classical result is almost identical to the former result deduced by considering the X-X interaction term in section S5.1.

(A similar classical treatment can be used to deduce the displacement under a nonlinear quadratic restoring force which, as mentioned before, well describes the first order correction for non-centrosymmetric media)

## Two-Level System (TLS)

The TLS was modeled using the von Neumann equation:

1. $\dot{\hat{\rho}}=\frac{i}{\hbar}\left[ \hat{\rho},\hat{H} \right]-\gamma\left( \begin{matrix} 0 & \rho_{01} \\ \rho_{10} & 0 \end{matrix} \right)$

where $\rho$ is the TLS density matrix, $\gamma$ is the dephasing rate associated with $T_{2}$ (set to the same value as in the AHO model), and $\hat{H}$ is the Hamiltonian. We note that $T_{1}$ terms can be ignored, as in room temperature $T_{1}$ for excitons in WSe_2_ is much larger than the dephasing time and the pulse temporal width. The Hamiltonian of a TLS can be written as:

$$\hat{H}=\left( \begin{matrix} 0 & -ea_{0}E\left( t \right) \\ -ea_{0}E\left( t \right) & \hbar\omega_{0} \end{matrix} \right)$$

where $\hbar\omega_{0}$ is the energy of the excited state, $e$ is the electron’s charge, $E\left( t \right)$ is the applied electrical field and $a_{0}$ is the exciton’s radius.

Taking the Fourier Transform of Eq. S6, we can then compute the off-orthogonal component $\rho_{01}$, which is proportional to the induced dipole moment and hence to the scattering. While the first order gives a solution similar to the harmonic oscillator, the second order yields:

1. $\delta\tilde{P}\left( \omega\right)\propto\delta\tilde{\rho}_{01}\left( \omega\right)+\delta\tilde{\rho}_{10}\left( \omega\right)\propto\frac{\tilde{E}\left( \omega\right)*((\frac{1}{\omega}\left( \tilde{E}\left( \omega\right)*\left( \frac{\tilde{E}\left( \omega\right)\left( \omega+i\gamma\right)}{\omega^{2}-\omega_{0}^{2}-2i\omega\gamma} \right) \right)}{\omega^{2}-\omega_{0}^{2}-2i\omega\gamma}$

In the given context, we observe that the weights on the electric field from Eq. S6 are not the same as those in Equation S5, though they are proportional to $E^{3}$. This difference results in a distinct FWM response when compared to the X-X term from Eq. S2 and the nearly identical AHO model. However, the PB term from Eq. S2 exhibits similar dynamics, as expected from a model that considers a single particle and does not incorporate interactions in a dense population.

## Control of nonlinear wave-mixing – multiphoton pathway interference

Multiphoton pathway interference is a phenomenon that occurs when electrons are excited through different multiphoton excitation paths by broadband femtosecond laser pulses. Electrons which are excited through different paths accumulate different phases and as a result interfere with one another. By the manipulation of the spectral phase of the driving pulse, one may control the nonlinear outcome of these interference effects.

## FWM destruction

Our control method, as highlighted in the main text, offers a constructive solution for reducing FWM generation while simultaneously preserving high peak power and a constant average power for the driving broadband pulse. Figure S8 presents a comparison between the FWM yield (Fig. S8.a), driving pulses’ spectral-phase (Fig. S8.b) and envelope (Fig. S8.c) of three different pulse shapes. The first pulse is an arctangent phased pulse (blue line) while the second pulse is a chirped pulse (red line), both of which generate the same FWM yield, 45-times weaker than the FWM generated by a third pulse, a transform-limited pulse (green line), and 127-times weaker than the maximal yield predicted by the AHO. While a chirped pulse stretches the pulse duration by 690% to 47 fs FWHM, the arctangent phased pulse causes the destruction of FWM yield while stretching the pulse by only 120% to 8.5 fs, while keeping the peak intensity relatively high. In essence, while a chirped pulse weakens all nonlinear contributions across the spectral bandwidth of the pulse, an arctangent-phased pulse can selectively modify the transient population in the excited state and as a result, weaken the nonlinear contribution of a chosen resonance while keeping the light-matter interaction intense.


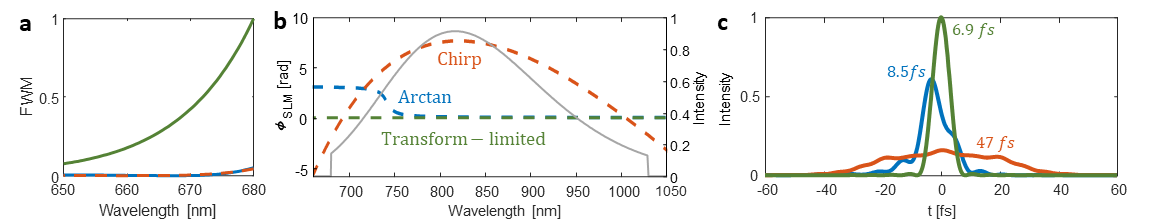


**Figure S8** **Destruction of the FWM yield**. a) Numerical comparison between two spectral phases which cause the same FWM reduction (47-times weaker than the FWM generated by a transform limited pulse). Transform-limited (green), Arctangent (blue) and chirped (red) spectral phases were applied to a Gaussian pulse. b) The spectral phases applied to a Gaussian pulse and, c) the temporal pulse shapes, respectively. While the Arctangent phase dramatically reduces the FWM, it maintains a relatively compressed pulse.

## Geometrical representation

To better understand the phenomenon of multiphoton pathway interference, we can visualize the nonlinear yield as a sum of all the possible multiphoton pathways leading to a final state. This can be represented through a geometrical path in the complex plane. Figures S9 and S10 present simulation predictions (second and third-order) for two different phase functions (chirp & arctangent). The predictions consist of a spectral representation of the phase function, a temporal profile of the driving pulse, a spectral analysis of the second and third-order nonlinear yield, and a geometrical phasor representation (caption) for both the instantaneous and non-instantaneous resonant cases (A1s exciton resonance).


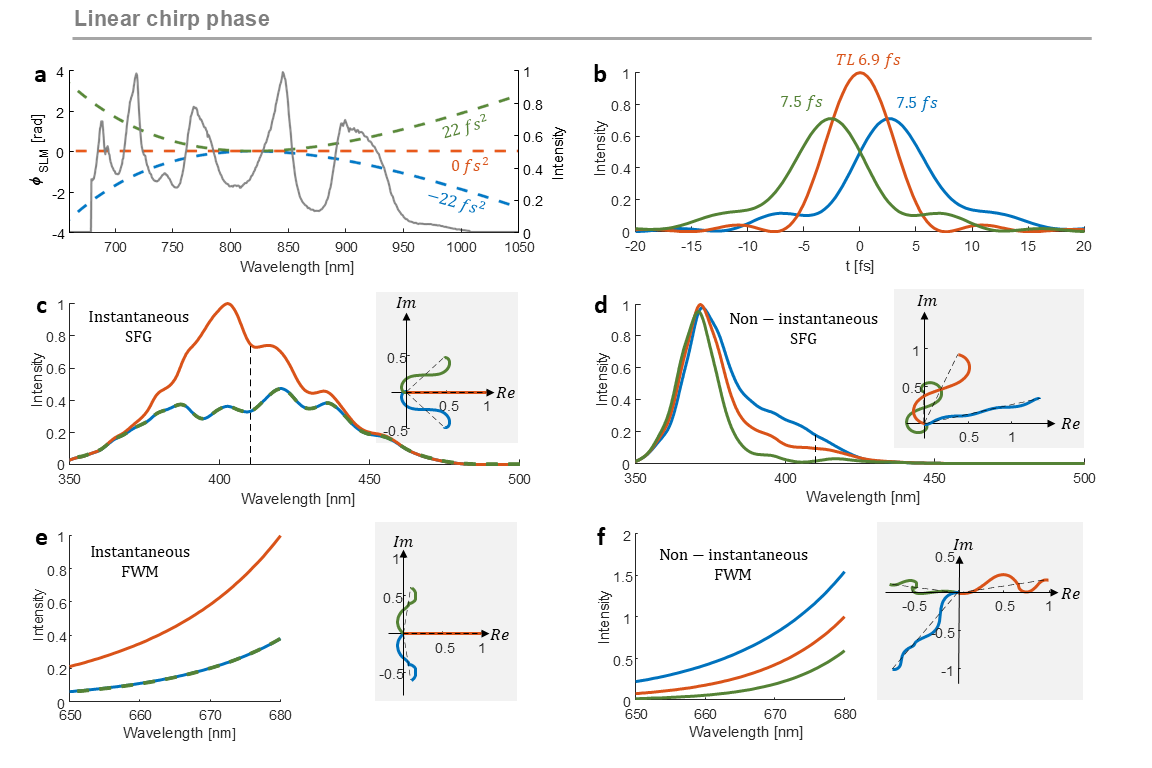


**Figure S9** simulations of the second & third-order nonlinear response to three different linear chirped pulse-shapes, $\beta=-22 fs$ (green), $\beta=-0 fs$ (orange), $\beta=22 fs$ (blue) . (a) spectral representation of the phase function plotted against the sub-10fs pulse spectrum. (b) temporal profile of the driving pulse for each phase, as well as the corresponding temporal FWHM. (c-f) a spectral analysis of the second (c-d) and third-order (e-f) nonlinear yield for an instantaneous interaction (c, e) as well as a noninstantaneous interaction (d, f) using the A1s exciton parameters. Insets: a geometrical representation of the third-order polarization phasor.


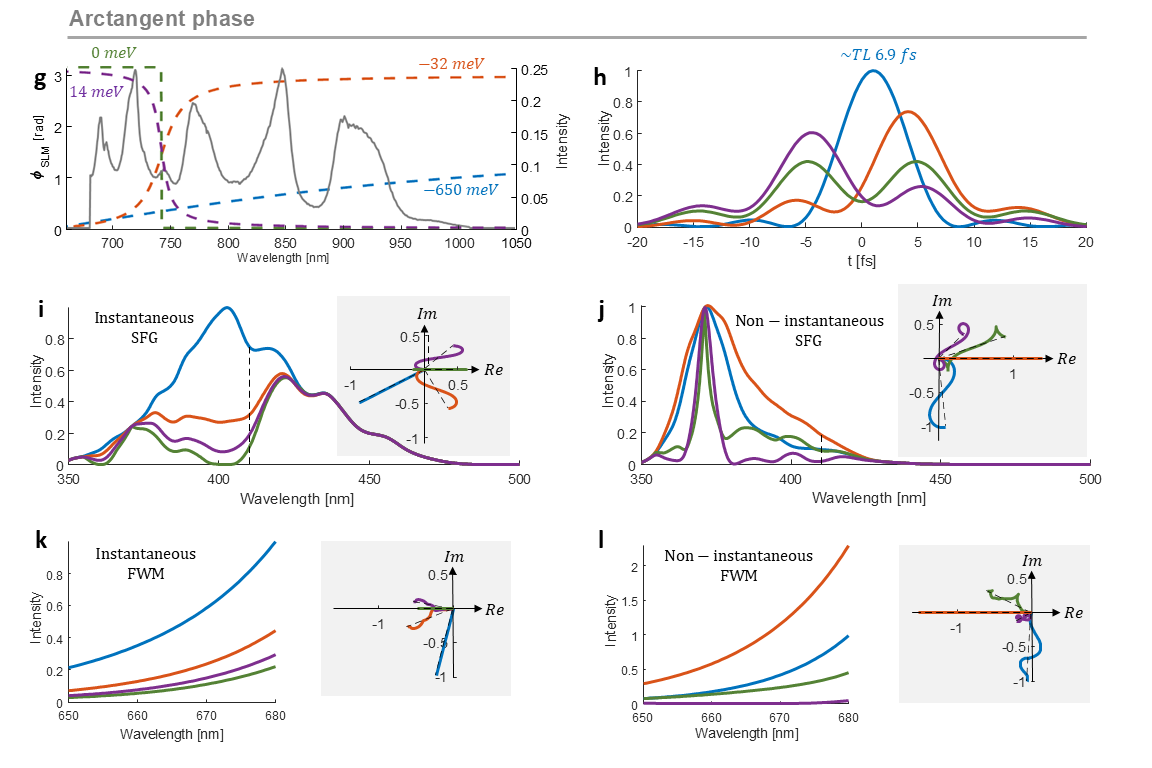


**Figure S10** simulations of the second & third-order nonlinear response to three different arctangent phased pulse-shapes, a nearly TL pulse ($\Gamma=-650 meV ,$blue), the optimal arctangent phase complementary to the A1s exciton resonance ($\Gamma=\gamma_{X_{1s}}=-32 meV$, orange), a $\pi$-step phase ($\Gamma=0 meV$, green), the phase causing the maximal nonlinear elimination ($\Gamma=14 meV$, purple). (a) spectral representation of the phase function plotted against the sub-10fs pulse spectrum. (b) temporal profile of the driving pulse for each phase. (c-f) a spectral analysis of the second (c-d) and third-order (e-f) nonlinear yield for an instantaneous interaction (c, e) as well as a noninstantaneous interaction (d, f) using the A1s exciton parameters. Insets: a geometrical representation of the third-order polarization phasor.

## The relative contribution of exciton-exciton interactions and Pauli blocking to FWM

The intensity of the FWM can be obtained by calculating the power density of the total dipole moment from Eq. S2 squared, which is the power density of the superposition of X-X and PB terms. To quantify the relative magnitude of $\hat{V}$ (the X-X coefficient) and $\hat{d}$ (the PB coefficient) to the overall FWM signal, we conducted a comparative analysis. We generated plots showing the FWM signal as a function of the varying strength ratios $f_{ratio}=\hat{V}/\hat{d}$, matching these qualitatively with our experimental results.

**
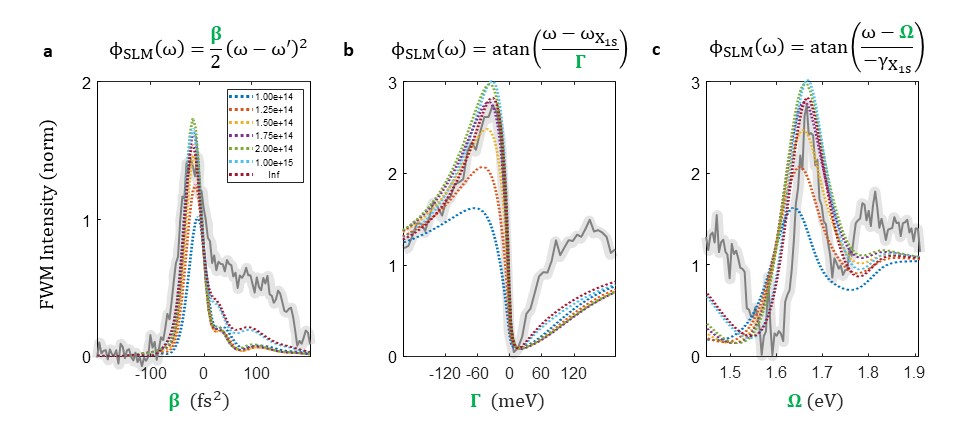
Figure S11 The relative contribution of X-X interactions and PB to FWM.** Our experimental FWM results (from Fig. 3 in the main text) are plotted against theoretical predictions of different ratios between the X-X to PB terms. (a) FWM intensity induced by a chirped pulse, with a varying GDD of $\beta$. (b-c) FWM intensity induced by an arctangent phased pulse. (b) The arctangent phase is centered around the A1s exciton resonance $\omega_{X_{1s}}$, while the linewidth $\Gamma$ is varied from negative to positive values. (c) The arctangent phase is set with a constant linewidth $-\gamma_{X_{1s}}$, and varying central frequency $\Omega$. All experimental results are scaled by a factor of 1.2 to fit the maximal enhancement of the X-X term.

Our findings indicate that the X-X interaction term dominates the FWM signal by a ratio factor larger than $2\times{10}^{14}$. This significant difference can also be justified from a unit analysis perspective. The PB term lacks a weight function D(ω) with units of ${10}^{15} sec^{-1}$ with respect to the XX term, explaining the observed disparity.

Below the ratio of $2\times{10}^{14}$ we see that the maximum calculated FWM does not match the position of the maximal signal measured in the experimental measurements. That is, the superposition between the two terms (X-X and PB) produces a redshifted maximal value in $\Omega$ scans and a significant broadening effect in the $\Gamma$ measurements which is inconsistent with measurements.

## Amplitude scan of the 2s arctangent phase

To further verify the superimposed arctangent phase centered at the 2s A-exciton energy and linewidth (presented in Fig. 4 in the main manuscript), we conducted an additional phase scan varying the amplitude of the second arctangent phase between 0 and 1.

**
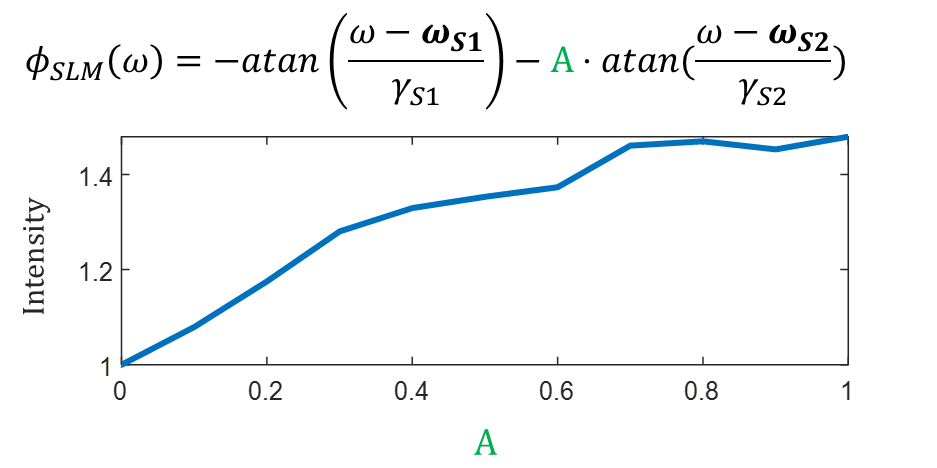
Figure S12 FWM intensity as a function of the amplitude A of the 2s arctangent phase.** The spectral phase $\phi_{SLM}$ of the pulse is modulated by varying A, which scales the superimposed arctangent phase centered at the 2s A-exciton energy and linewidth. This modulation is applied in addition to an arctangent phase centered at the 1s A-exciton energy and linewidth.

# References

| [1] | N. Dudovich, D. Oron and Y. Silberberg, "Single-pulse coherently controlled nonlinear Raman spectroscopy and microscopy," *Nature,* vol. 418, p. 512–514, 2002. |
| --- | --- |
| [2] | W. Min, S. Lu, M. Rueckel, G. R. Holtom and X. S. Xie, "Near-Degenerate Four-Wave-Mixing Microscopy," *Nano Lett.,* vol. 9, p. 2423–2426, June 2009. |
| [3] | H. Suchowski, K. O’Brien, Z. J. Wong, A. Salandrino, X. Yin and X. Zhang, "Phase Mismatch–Free Nonlinear Propagation in Optical Zero-Index Materials," *Science,* vol. 342, p. 1223–1226, December 2013. |
| [4] | V. Kravtsov, R. Ulbricht, J. M. Atkin and M. B. Raschke, "Plasmonic nanofocused four-wave mixing for femtosecond near-field imaging," *Nature Nanotechnology,* vol. 11, p. 459–464, May 2016. |
| [5] | E. Bahar, U. Arieli, M. V. Stern and H. Suchowski, "Unlocking Coherent Control of Ultrafast Plasmonic Interaction," *Laser & Photonics Reviews,* vol. 16, p. 2100467, 2022. |
| [6] | N. Dudovich, B. Dayan, S. M. Gallagher Faeder and Y. Silberberg, "Transform-Limited Pulses Are Not Optimal for Resonant Multiphoton Transitions," *Phys. Rev. Lett.,* vol. 86, no. 1, p. 47–50, January 2001. |
| [7] | C. Trovatello, F. Katsch, N. J. Borys, M. Selig, K. Yao, R. Borrego-Varillas, F. Scotognella, I. Kriegel, A. Yan, A. Zettl, P. J. Schuck, A. Knorr, G. Cerullo and S. D. Conte, "The ultrafast onset of exciton formation in 2D semiconductors," *Nature Communications,* vol. 11, p. 5277, 2020. |
| [8] | F. Katsch, M. Selig and A. Knorr, "Exciton-Scattering-Induced Dephasing in Two-Dimensional Semiconductors," *PRL,* vol. 124, p. 257402, June 2020. |
| [9] | F. Katsch, M. Selig and A. Knorr, "Theory of coherent pump-probe spectroscopy in monolayer transition metal dichalcogenides," *2D Materials,* vol. 7, p. 015021, 2019. |
| [10] | R. Boyd, Nonlinear optics, San, Diego: Academic Press, 2003. |
